# Inside Ceramics and Between MgO Grains: Solid‐State Synthesis of Intergranular Semiconducting or Magnetic Spinels

**DOI:** 10.1002/smtd.202400715

**Published:** 2024-10-29

**Authors:** Thomas Schwab, Korbinian Aicher, Gregor A. Zickler, Michael Reissner, Oliver Diwald

**Affiliations:** ^1^ Department of Chemistry and Physics of Materials Paris‐Lodron University Salzburg Jakob‐Haringer‐Straße 2a Salzburg A‐5020 Austria; ^2^ Institute of Solid State Physics TU Wien Wiedner Hauptstraße 8–10 Vienna A‐1040 Austria

**Keywords:** grain boundary resistivity, intergranular films, metal oxide ceramics, segregation, spinel phase

## Abstract

Configurations of composite metal oxide nanoparticles are typically far off their thermodynamic equilibrium state. As such they represent a versatile but so far overlooked source material for the intergranular solid‐state chemistry inside ceramics. Here, it is demonstrated how the admixture of Fe^3+^ and In^3+^ ions to MgO nanoparticles, as achieved by flame spray pyrolysis, can be used to engage ion exsolution, phase separation, and subsequent spinel formation inside the network of diamagnetic and insulating MgO grains. Extremely high uniformity in the distribution of intergranular ferrimagnetic MgFe_2_O_4_ films and grains with resulting magnetic coercivity values that depend on the nanoparticles’ initial Fe^3+^ concentration is achieved. Moreover, percolating networks of semiconducting MgIn_2_O_4_ are derived from MgO nanoparticles with admixtures of 20 at% In^3+^ that gives rise to an enhancement of dc conductivity values by more than five orders of magnitude in comparison to the insulating MgO host. The here presented approach is general and applicable to the synthesis of a variety of functional spinel nanostructures embedded inside ceramic matrices. Nanoparticle loading with aliovalent impurity ions, the level of nanoparticle powder density after compaction, and sintering temperature are key parameters for this novel type of solid‐state chemistry in between the host grains.

## Graphical abstract

Intergranular regions between composite metal oxide grains serve as a novel reaction space for the synthesis of functional nanostructures during ceramic sintering. Trivalent Fe^3+^ and In^3+^ ions trapped within MgO grains react with MgO to form spinel phases with ferrimagnetic or semiconducting properties, respectively. Here, it is demonstrated that intergranular solid‐state chemistry is a versatile approach to produce electroceramics, catalysts, and magnetic devices.

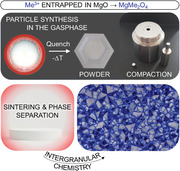

## 1 Introduction

Within ceramics, grain boundaries, intergranular films, and regions that differ in structure and composition from the surrounding grains represent an extremely exciting opportunity region for materials chemistry. With different types of interfaces, such regions accumulate intrinsic and extrinsic defects and usually exhibit altered reactivity or electrochemical potentials. They host important functional properties as a result of altered values for ion diffusivity, electrical resistivity, or magnetism compared to the grains' bulk.^[^ ^1^, ^2^, ^3^, ^4^ ^]^

So far, the altered structure and composition of intergranular regions and their relation to space charge regions have been analyzed.^[^ ^5^, ^6^, ^7^, ^8^ ^]^ From another perspective, research to exploit the chemical reactivity of intergranular regions as key elements of a ceramic microstructure will advance our understanding about chemistry in confined space and may point to new directions for the design of novel nanostructures. However, associated efforts require control of the material situation prior to sintering, i.e., prior to the thermally activated mass transport of reactants towards or within the reaction zone and confined by the boundary grains. Powders of composite metal oxide nanoparticles with non‐equilibrium configuration^[^ ^9^, ^10^, ^11^ ^]^ represent a well‐suited starting point in this respect.

**Figure 1:**
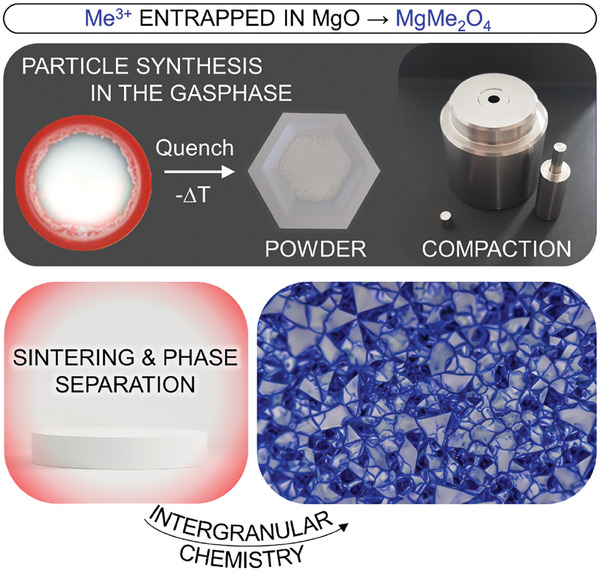
Composite metal oxide nanoparticles that nucleate and grow at high temperatures within microseconds and subsequently become quenched to room temperature (top) are metastable. After room temperature processing and compaction resulting packed particle systems can then host intergranular solid‐state chemistry during sintering (bottom).

This study demonstrates for the first time how the use of composite model particle systems can reveal key details of the sinter‐induced intergranular chemistry that ultimately determines the evolution of microstructure in functional ceramics. We will provide previously unavailable insights into the formation of ferromagnetism or electronic conductivity in MgO‐based materials. Here, MgO serves as a diamagnetic and insulating ceramic host and has no special function apart from the structural properties itself. We will show that metal oxide nanoparticles, which nucleate and grow within microseconds and at high temperatures during gas phase synthesis, are a well‐suited starting point for subsequent powder compaction and sintering. Sintering, in turn, activates the intergranular chemistry inside ceramics (**Figure** **1**). Methods such as flame spray pyrolysis^[^ ^12^, ^13^, ^14^, ^15^ ^]^ (FSP) and gas phase synthesis approaches in general^[^ ^11^ ^]^ are highly instrumental to incorporate selected individual impurity ions into the lattice of the host particle.^[^ ^11^, ^16^ ^]^ The average residence time of the growing solid particles within the hot reaction zone is short and thus a variety of solid non‐equilibrium configurations can emerge from subsequent particle quenching to room temperature. Recently, an advanced flame‐aerosol process has been developed to produce metastable nanoshells^[^ ^17^ ^]^ with catalytic properties that represent a significant extension of the materials space. Such approaches exceed the opportunities for materials design of equilibrium synthesis processes by far.^[^ ^9^, ^18^ ^]^

With this work, we take the fabrication process of nanoparticle‐based ceramics a decisive step forward and focus on the design of functional intergranular loadings that are spread through an essentially inert, diamagnetic, and insulating network of MgO grains (Figure 1). We show for functional spinel precipitates how their dispersion and connectivity – in the form of magnesium ferrite MgFe_2_O_4_ and magnesium indate MgIn_2_O_4_ – can be easily controlled via selection of the mixing concentration and well‐defined sintering protocols. More specifically, we apply for the first time the concept of phase separation of nanocrystalline Me^3+^/MgO particles, which in terms of chemical composition are uniformly distributed, to generate functional spinel structures with the generic formula AB_2_O_4_.^[^ ^18^, ^19^ ^]^ In bulk form, the latter ceramics are also of great interest for materials for optical applications,^[^ ^20^ ^]^ as magnetic materials^[^ ^21^, ^22^, ^23^, ^24^, ^25^, ^26^ ^]^ or as components for transparent and conductive oxides,^[^ ^27^, ^28^, ^29^, ^30^ ^]^ just to name a few.

## 2 Results and Discussion

### 2.1 From Nanoparticle Compacts to Phase Separation Induced Intergranular Chemistry

This work underlines the high potential of composite metal oxide nanoparticles as versatile nanocrystalline building blocks and precursors for subsequent microstructure engineering through sintering. FSP as a valuable alternative to chemical vapor synthesis (CVS)^[^ ^16^ ^]^ allows for the scalable synthesis of particles with comparable purity and structural definition compared to CVS.^[^ ^31^, ^32^ ^]^ For impurity admixtures with concentrations of up to 10 at% for Fe^3+^ and 20 at% for In^3+^, we obtained crystalline particles that reveal the MgO specific cubic structure in the respective X‐ray diffraction (XRD) patterns (Figure S2.1 and Table S2.1, Supporting Information). Moreover, the narrow particle size distributions as determined from particle counting and transmission electron microscopy (TEM) data analysis (Figures S2.2, S2.3 and Table S2.2, Supporting Information), is perfectly consistent with the XRD derived average crystallite domain sizes of ≈5–6 nm.

**Figure 2:**
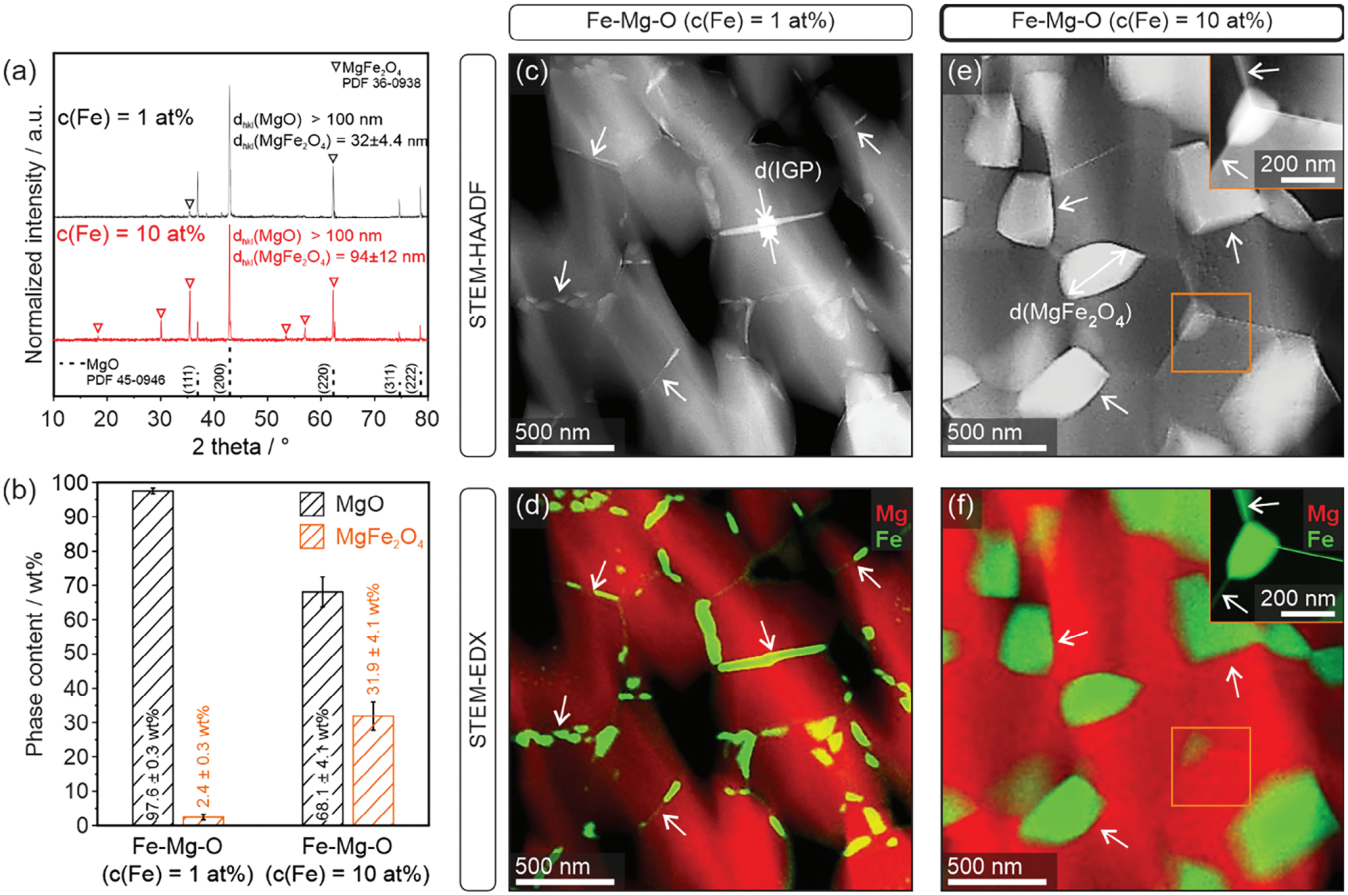
a) XRD pattern and b) phase analysis results from Rietveld refinement^[^ ^33^ ^]^ of Fe‐Mg‐O nanoparticle compacts with Fe concentrations of 1 at% and 10 at% after sintering at 1373 K; TEM analysis (c–f) clearly shows that phase separation and Fe‐segregation has occurred in Fe─Mg─O ceramics with nominal Fe‐concentrations of 1 at% (c,d) and 10 at% (e,f). Scanning transmission electron miroscopy high angular annular dark field (STEM‐HAADF) images (c,e) provide information about the compositional contrast. Enhanced contrast is attributed to Fe‐segregation between MgO‐based grains (indicated by arrows) and confirmed by scanning transmission electron microscopic energy dispersive X‐ray spectroscopy (STEM‐EDX) intensity maps (d,f). The inset in (f) shows Fe‐rich triple‐junctions between MgO‐based grains.

Sintering of compacts of Fe containing MgO nanoparticles with Fe concentrations of 1 and 10 at% at 1373 K produces the magnesium ferrite (MgFe_2_O_4_) spinel phase (Equation 1 and **Figure** **2**a) with weight fractions of 2.4 and 32 wt.% (Figure 2b), respectively.

(1) $$\mathrm{MgO}+2Fe^{3+}+3O^{2-}\to \mathrm{MgF}e_{2}O_{4}$$

Crystallite domain sizes determined from the refinement of the entire pattern^[^ ^33^ ^]^ revealed that the MgO grains adopt sizes >100 nm, while the MgFe_2_O_4_ domains remain in the nanocrystalline size regime with average values of 32 and 94 nm for samples with Fe concentrations of 1 and 10 at%, respectively.

High angular annular dark field (HAADF) images (Figure 2c,e) reveal compositional contrast and provide information about the local distribution of the exsolved iron. According to Selected Area Electron Diffraction (SAED) analysis these intergranular features can be unambiguously attributed to the magnesium ferrite phase (Figure S2.4, Supporting Information). For low Fe concentrations (Figure 2c,d) relatively thin Fe‐rich intergranular films (d = 22 ± 15 nm) become entrapped between the MgO based grains. They seem to effectively fill the spaces between the individual grains, but do not decorate the free pore surfaces.^[^ ^4^ ^]^

### 2.2 Defect Chemistry of Fe^3+^ Ions and Intergranular Magnetism

During gas phase synthesis iron ions become entrapped within the growing magnesium oxide nanocrystals. Prior to annealing related configurations correspond to a non‐equilibrium state. Fe^3+^ ions reside in isolated octahedral positions of the cationic sublattice. Thermal treatment at 873 K and higher was found to decrease the local symmetry around them, as evidenced in earlier X‐ray absorption spectroscopy measurements.^[^ ^32^, ^34^ ^]^

**Figure 3:**
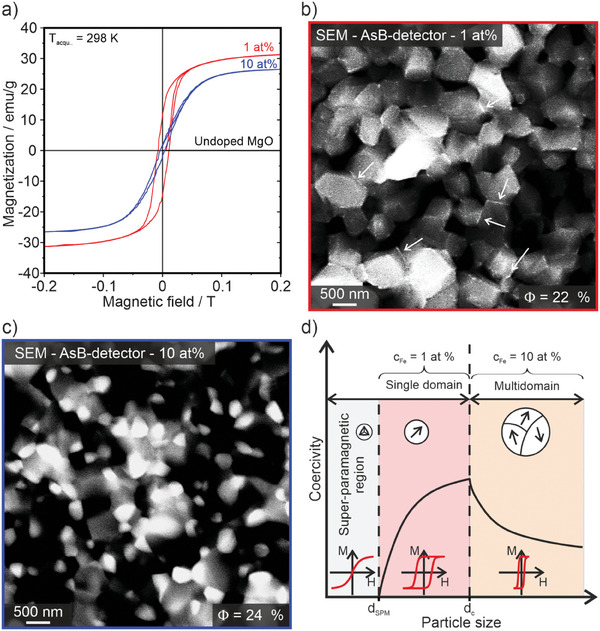
a) Magnetic hysteresis loops and saturation magnetization values as determined by room temperature vibrating sample magnetometer (VSM) measurement for an iron free and diamagnetic MgO ceramic (black line), for a Fe─Mg─O ceramic with Fe concentrations of 1 at% (red line) and 10 at% (blue line). All samples were sintered at 1373 K. The measured magnetizations (Table 1) were related to the respective weight fractions of the magnetic MgFe_2_O_4_ phase as concluded from phase analysis (Figure 2b). Figure 3b,c shows representative scanning electron microscopy (SEM) images of the two samples with comparable porosity values. The scheme in d provides a tentative explanation for the significant difference in the coercivity values for the two samples shown in (a, see text for further information).

This decrease in local symmetry around the aliovalent Fe^3+^ impurities is attributed to their clustering with cation vacancies for electrostatic compensation to form dimer and trimer complexes. At iron concentrations above 1 at% the contribution of particle coarsening and defect clustering favors the formation of dimers, trimers, and larger impurity cation vacancy clusters.^[^ ^32^, ^34^ ^]^ Associated grain growth proceeds along the spinel motif of the MgFe_2_O_4_ nuclei that initiate the formation of the intergranular magnesium ferrite phase (Equation 1 and Figure 2c–f). Magnesium ferrite crystallizes in the spinel structure and shows ferrimagnetic ordering with typical values of σ_m_ = 21 emu per gram.^[^ ^35^, ^36^, ^37^ ^]^ For the characterization of the intergranular magnesium ferrite we measured the magnetic hysteresis loops (**Figure** **3**a and **Table** **1**). The signal of the magnetization of the pure and diamagnetic host MgO (black line) is below the detectable limit of the instrument. As the shape of the magnetic hysteresis loops is typically determined by domain states (Figure 3d), we can infer from the results of the magnesium ferrite containing samples with 1 and 10 at% Fe on the existence of at least two different ferrimagnetic grain sizes: a fine single domain fraction that is associated with larger coercivity values (red line in Figure 3a) and a coarsened multidomain fraction (Figure 3d) with smaller coercivity (blue line in Figure 3a).

**Table 1:** Summary of the saturation magnetization, remnant magnetization, and coercivity of the Fe─Mg─O ceramics with nominal Fe‐concentrations of 1 and 10 at%, sintered at 1373 K.

	Saturation magnetization M_S_/emu∙g^−1^	Remanent magnetization M_R_/emu∙g^−1^	Coercivity H_C_/mT
MgFe_2_O_4_ in MgO 1 at% Fe	35	11.7	8.1
MgFe_2_O_4_ in MgO 10 at% Fe	28	2.0	3.7

Loading the intergranular regions with different concentrations of magnesium ferrite impacts the observed magnetic parameters of the soft magnetic phase. For pellets with low concentrations (1 at% Fe), where we detected with element‐resolved electron microscopy thin (22 ± 15 nm) magnesium ferrite layers between the individual grains (Figure 3b), we observe a less soft magnetic behavior and larger coercivity values (Figure 3a) as compared to 10 at% Fe samples with larger magnesium ferrite grains (d > 250 nm, Figure 2e,f).

The room temperature saturation magnetization M_S_ = 35 emu g^−1^ of the 1 at% Fe sample is clearly comparable to other nanosized magnesium ferrite samples^[^ ^38^, ^39^, ^40^ ^]^ and exceeds respective values for the 10 at% sample (M_S_ = 28 emu g^−1^, Figure S2.5 and Table S2.3, Supporting Information).

Recent interest in nanosized spinel type ferrites arises from their suitability as key components in electronics, for magnetic storage devices, ferrofluid technology, or for environmental and medicinal applications.^[^ ^41^, ^42^, ^43^ ^]^ The here presented nanostructured intergranular films and grains with their variable ferrimagnetic properties are enclosed in a diamagnetic host matrix. In comparison to traditional nanoparticle synthesis approaches very different synthesis parameters, such as the initial iron content of the nanoparticle grains, the powder density, as it can be adjusted by compaction, as well as temperature and time of annealing to generate phase separation, apply. Also, the wetting behavior of the emergent spinel phase on the coarsening MgO grains plays a decisive role and determines the distribution of the ferrimagnetic inclusions between the grains and pores (Figure 2). The dependance of the coercivity values on the magnesium ferrite distribution over the MgO microstructure is here tentatively explained by the presence of single‐ and multidomain regions. Their Fe concentration dependent emergence as well as the determination of a critical threshold for the transition from the monodomain to the multidomain states requires a more detailed and systematic analysis.

### 2.3 Ion Exsolution and Emergence of Percolating Semiconducting Network

The introduction of electronic conductivity to a network of insulating grains requires, however, substantially higher amounts of functional admixtures. This is demonstrated for gas phase‐based admixture of In^3+^ ions to growing MgO nanoparticles with indium concentrations of up to 20 at%. Even at such a loading there is no evidence for a pronounced separation and crystallization of a secondary indium oxide related phase in the XRD patterns of the as‐synthesized nanoparticle powders (Figure S2.1, Supporting Information).

Non‐equilibrium In─Mg─O nanoparticle powders can be produced by chemical gas phase synthesis via injection of an indium containing organic precursor into the magnesium combustion flame^[^ ^31^ ^]^ or, alternatively, by FSP (this work). Subsequent annealing drives impurity segregation and, after powder densification by compaction, is instrumental to engineer particle‐particle interfaces, grain boundaries or intergranular regions (Figure 1).

**Figure 4:**
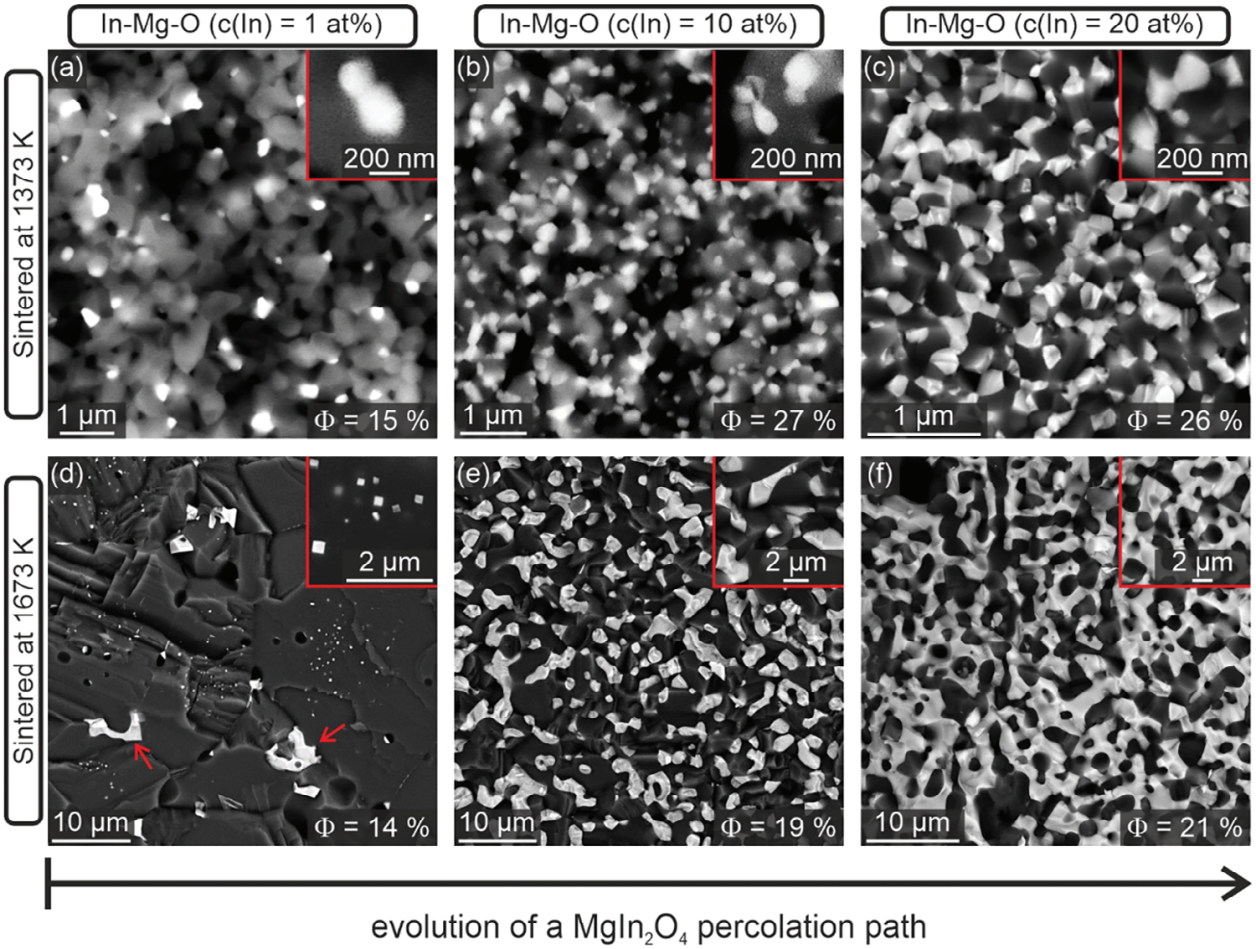
Series of SEM images of In─Mg─O nanoparticle compacts after sintering at 1373 K (top row) and 1673 K (bottom row), which illustrate the impact of indium loading from 1 at% (a,d), 10 at% (b,e), and 20 at% (c,f) on the distribution of MgIn_2_O_4_.

In the attempt to engage the reaction between MgO and In_2_O_3_ to form MgIn_2_O_4_ spinel inside the MgO based matrix (Equation 2),^[^ ^44^, ^45^, ^46^, ^47^ ^]^

(2) $$\mathrm{MgO}+\;In_{2}O_{3}\to \mathrm{MgI}n_{2}O_{4}$$

we chose higher indium concentrations (up to 20 at%) and sintering temperatures, i.e., 1673 K, as compared to MgFe_2_O_4_ (**Figure** **4**).

Figure 4 clearly shows the local distribution of indium that changes from isolated grains at low concentrations (Figure 4a,d; Figure S2.4, Supporting Information) – some of which still adopt an In_2_O_3_‐specific cubic shape – to a continuous strand‐like indium‐containing network in samples with concentrations of 20 at% In (Figure 4c,f). The bright areas in the SEM images correspond to the indium‐containing precipitates in contrast to the Mg‐dominated substrate in dark. All the samples investigated have residual porosities in the range between 15% and 27%, which explains why corresponding pellets remain opaque while showing enhanced conductivity (see below).

**Figure 5:**
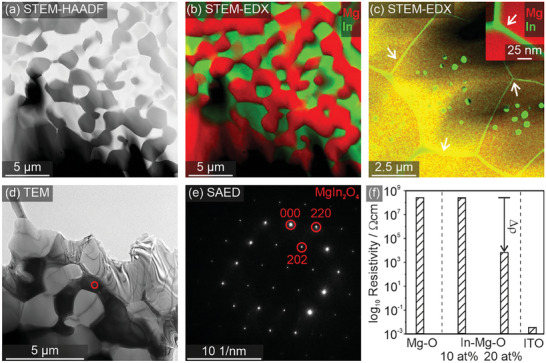
TEM data related to an In─Mg─O ceramic with 20 at% indium and a residual porosity of 21%. STEM‐EDX mapping (a–c) reveals µm thick and In rich strands (green) in the percolation network (a–c). TEM images taken at larger magnifications do also show thinner intergranular films with thicknesses between 5 and 30 nm (inset in c) and particulate precipitates. SAED data acquired from the crystalline region (d,e) confirm its spinel‐type MgIn_2_O_4_ structure. Results from 4 point‐probe resistivity measurements at room temperature are plotted in (f).

The solid‐state reaction between MgO and In_2_O_3_ to form a semiconducting MgIn_2_O_4_ phase (Equation 2) requires reaction temperatures above 1373 K (Figure S2.7, Supporting Information). For this reason, we discuss here Figures 4f and **5** results that were obtained on ceramic pellets after sintering at 1673 K in O_2_ atmosphere. The STEM‐HAADF and STEM‐EDX images in **Figure** **5**a–c show percolating intergranular networks of an indium‐containing phase. In agreement with the XRD results revealing the MgIn_2_O_4_ spinel as exclusive crystalline phase with indium, TEM in conjunction with selected area electron diffraction SAED confirms crystallinity and phase composition of the intergranular region (Figure 5d,e).

The emergence of a percolating MgIn_2_O_4_ network inside the insulating granular MgO host matrix strongly impacts the compact's integral electronic resistivity. While a sample with 10 at% indium (In) is not yet able to form a continuous and percolating network of semiconducting MgIn_2_O_4_, for a 20 at% sample the amount of added indium (In) is sufficient. Only for this sample and after sintering at 1673 K the resulting electronic resistivity value decreases by more than 4 orders of magnitude. Moreover, corresponding conductivity value exceeds that of a thin MgIn_2_O_4_ film prepared by magnetron sputtering by three orders of magnitude.^[^ ^27^ ^]^ A compact prepared from an indium tin oxide (ITO) ceramic powder, which was processed in the same way as the nanoparticle powders, is taken as a reference and shows an electronic resistivity that remains six orders of magnitude lower than the nanoparticle compact (Figure 5f).

## 3 Conclusion

Intergranular regions inside compacts of composite metal oxide nanoparticles represent a previously overlooked and novel reaction space for the solid‐state synthesis of nanostructures. During ceramic sintering trivalent exsolute ions that were initially trapped inside the MgO‐based grains react with Mg^2+^ and O^2−^ ions to generate functional spinel phases of the MgMe_2_O_4_ type. As versatile nanocrystalline building blocks and precursors such composite nanoparticles can be employed for subsequent microstructure engineering through sintering. With Fe^3+^ or In^3+^ ion exsolution as a key step during sintering, we demonstrate how ferrimagnetism or semiconductivity can be added to the insulating and diamagnetic microstructure of the MgO host. Many different impurity ions can be easily admixed to the MgO host during gas phase synthesis to generate a large variety of intergranular and functional spinel structures. There exist many application fields for materials with functional spinel structures to be utilized as (photo)electrodes, electroceramics, catalysts, or magnetic devices. Moreover, we believe that the principle of utilizing the phase separation behavior of non‐equilibrium solids for the design of functional intergranular regions goes beyond spinels and can be applied to other metal oxide structures as well.

## 4 Experimental Section

### Nanoparticle Synthesis

The self‐constructed flame spray apparatus is described in the Supporting Information and in Refs. [11, 16]. The synthesis and preparation of the precursor solutions of the Me─Mg─O nanoparticles is based on the synthesis protocol of Schwab et al.^[^ ^4^ ^]^ For the precursor preparation of MgO without admixtures, the Mg‐concentration was set to 0.75 mol∙L^−1^ by dissolving 16.1 g of magnesium acetate tetrahydrate [Mg(C_2_H_3_O_2_)_2_∙4H_2_O, 99% Strem Chemicals] in 33.4 mL of 2‐ethylhexanoic acid (2‐EHA, >99%, Sigma–Aldrich/Merck) and 20 ml of methanol (≥ 99.8%, Sigma–Aldrich/Merck) under vigorous stirring at room temperature and filled with a complementary volume of xylene (≥98.5%, VWR Chemicals) to fill a 100 mL batch.

For the synthesis of Fe_x_‐Mg_1‐x_‐O and In_x_‐Mg_1‐x_‐O nanoparticles the metal concentration within the Fe‐ and In‐precursor solution was set to 0.34 mol∙L^−1^ by dissolving either ferrocene [Fe(C_5_H_5_)_2_, 98%, Sigma–Aldrich/Merck] in xylene or indium acetylacetonate [In(C_5_H_7_O_2_)_2_, 98%, Strem Chemicals] in toluene under vigorous stirring at room temperature. (Table S1.2, Supporting Information) Respective volume ratios of Fe‐ or In‐precursor solutions were mixed with the Mg‐precursor solution to achieve the desired Me^3+^ impurity ion concentrations of 1, 10, and 20 at%.

### Ceramic Processing

Powder compaction was performed via uniaxial pressing of the nanoparticle powder at room temperature under ambient conditions, resulting in regular disk‐shaped specimens. A defined mass of powder (m  =  150 ± 10 mg) was transferred into the cavity (d = 13 mm) of a compaction tool (FTIR Pellet Dies, Specac) and uniaxially compressed (p = 74 MPa) with a hydraulic press (Atlas Manual Hydraulic Press 15T, Specac). (Further details in the Supporting Information)

Pressureless sintering of the green bodies was performed within a horizontally operated high temperature ceramic tube furnace (Nabertherm RHT80‐300/16) that allows the addition of defined gas flows.

### Characterization Methods

*X‐ray diffraction*: Powder XRD data were collected at room temperature in coupled Theta–Theta mode on a Bruker D8 Advance with DaVinci design diffractometer, having a goniometer radius of 280 mm and equipped with a fast‐solid‐state Lynxeye detector and an automatic sample changer. Powder samples and ceramic specimen, dry‐ground in a porcelain mortar, were prepared on a zero‐background sample holder made of single‐crystalline silicon. Data acquisition was done using Cu Kα1,2 radiation (λ = 154 pm) between 5° and 80.5° 2Θ with a step size of 0.02° and opened divergence and anti‐scatter slits at 0.3° and 4°, respectively. A primary and secondary side 2.5° Soller slit was used to minimize axial divergence and a detector window opening angle of 2.93° was chosen. Data handling and qualitative phase analysis was performed with the Bruker software DIFFRAC.EVA V2.1. For quantitative phase analysis, the Rietveld program TOPAS 4.2 (Bruker 2012) was used by modeling the intrinsic peak shape of the Bragg peaks using the fundamental parameter approach. Crystallite domain sizes were derived from the integral reflection width by applying the Scherrer equation^[^ ^48^ ^]^ either to the main diffraction feature or using a whole pattern refinement with the Rietveld method^[^ ^33^ ^]^ for qualitative or quantitative phase analysis, respectively.

*Transmission electron microscopy*: TEM data were acquired using a JEOL JEM‐F200 transmission electron microscope operating at 200 kV equipped with a cold field emission electron source and a large windowless JEOL Centurio EDX (Energy Dispersive X‐ray emission) detector (100 mm^2^, 0.97 srad, energy resolution <133 eV @ MnKα) for local composition analysis. STEM images, such as secondary and back scattered electron or HAADF images showing z‐contrast as well as EDX intensity maps were acquired in STEM mode. A typical beam current of 0.1 nA and a beam diameter of 0.16 nm during an acquisition time between 10 and 30 min was applied. Maps were obtained by signal integration of counts over the Mg Kα transition line for Mg (integration: 1.16–1.34 keV) and respective Kα or Lα transition lines of investigated admixture species (see Table S1.3, Supporting Information). TEM as well as high resolution TEM (HR‐TEM) and SAED images to access morphological and structural information were recorded using a TVIPS F216 2k by 2k CMOS camera. Evaluation of images acquired during TEM analysis was performed with either ImageJ (V1.52a) or the EM Measure software from TVIPS. Details about the TEM specimen preparation are provided in the Supporting Information.

*Scanning electron microscopy*: Imaging of ceramic fracture surfaces was performed with a scanning electron microscope (SEM, Zeiss Ultra Plus 55) equipped with a field‐emission gun, Gemini lenses, and an Oxford Instruments EDX‐Silicon drift detector (50 mm^2^, energy resolution < 127 eV @ MnKα). Secondary electron (SE) images providing topological information and backscattered electron (BSE) images showing z‐contrast were acquired at short working distances of ≈3 mm and an accelerating voltage between 5 kV‐20 kV, with an InLens SE and AsB (Angular selective Backscatter) detector, respectively.

*Conductivity measurements*: Resistivity investigations at room temperature were carried out with the Signatone Pro4 – four‐point probe (4PP) resistivity measurement setup from Lucas Labs, equipped with a Keithley 2400 sourcemeter. Samples were contacted by using a commercially available probe‐head (tip spacing: 1.02 mm, tip radius: 0.041 mm) consisting of four thin collinear arranged and equally spaced point electrodes.

*Vibrating sample magnetometry*: Magnetization measurements at room temperature were performed at TU Wien with a 9T Physical Property Measurement System (PPMS) with Vibrating Sample Magnetometer (VSM) option from Quantum Design. Hysteresis loops in the magnetic field range ± 9 T with a sweep rate of 5 mT·s^−1^ to obtain saturation magnetization and in the field of ± 0,2 T with a sweep rate of 0.1 mT·min^−1^ in the range ± 10 mT to get information about the coercive field were performed.

## Conflict of Interest

The authors declare no conflict of interest.

## Supporting information



Supporting Information

## Data Availability

The data that support the findings of this study are available from the corresponding author upon reasonable request.
