# Supplementary material for: Inside Ceramics and Between MgO Grains: Solid‐State Synthesis of Intergranular Semiconducting or Magnetic Spinels
Source: Small Methods. 2024 Oct 29;9(1):2400715. doi: 10.1002/smtd.202400715 (PMC11740943; doi:10.1002/smtd.202400715)
Supplement: Supplementary file 1 — Supporting Information [file SMTD-9-2400715-s001.docx]

**Inside Ceramics and between MgO Grains: Solid-State Synthesis of Intergranular Semiconducting or Magnetic Spinels**

*Thomas Schwab^a,#^, Korbinian Aicher^a,#^, Gregor A. Zickler^a^, Michael Reissner^b^ and Oliver Diwald^a,*^*

^a^ Department of Chemistry and Physics of Materials,

Paris-Lodron University Salzburg, Jakob-Haringer-Straße 2a,

A-5020 Salzburg, Austria

^b^ Institute of Solid State Physics, TU Wien, Wiedner Hauptstraße 8-10,

A-1040 Vienna, Austria

E-mail: oliver.diwald@plus.ac.at

**Keywords**: intergranular films, segregation, metal oxide ceramics, grain boundary resistivity, spinel phase;

Table of contents

1 Supporting Information – Experimental 2

1.1 Nanoparticle synthesis 2

1.1.1 Flame spray pyrolysis setup and parameters 2

1.1.2 Precursor solutions 3

1.2 Ceramic manufacturing 3

1.3 Characterization methods 4

2 Supporting Information – Results 6

2.1 Me-Mg-O nanoparticle powder characterization 6

2.1.1 X-ray diffraction (XRD) 6

2.1.2 Transmission electron microscopy (TEM) 7

2.2 Sintered ceramic characterization 9

# Supporting Information – Experimental

## Nanoparticle synthesis

### Flame spray pyrolysis setup and parameters

A generalized scheme of the flame spray pyrolysis (FSP) setup as well as synthesis-related production parameters are provided in **Figure S1.1** and **Table S1.1**, respectively.


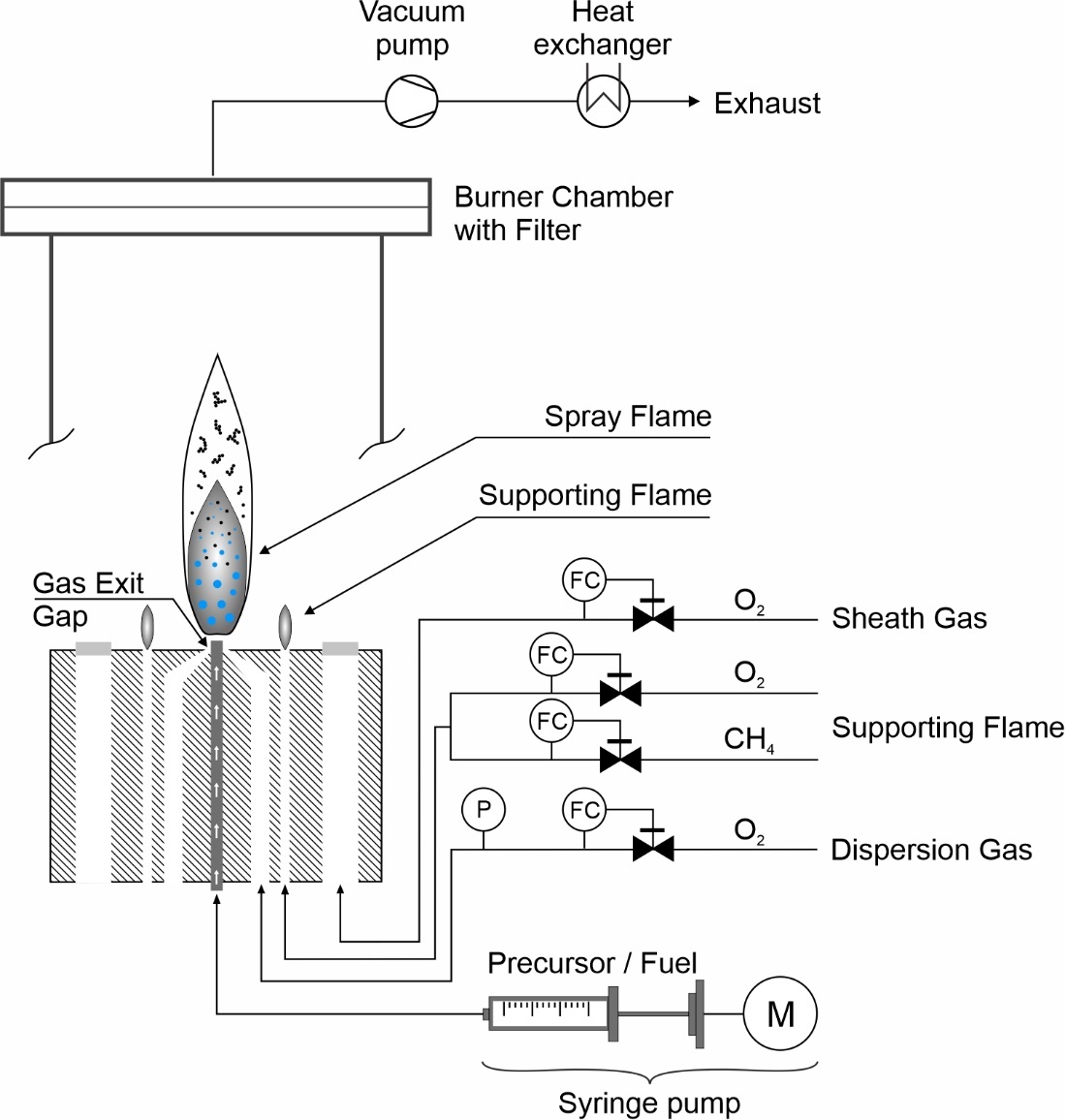


**Figure S1.1:** Schematic representation of the in-house built FSP reactor setup for the synthesis of Me-Mg-O (Me = Fe, In) nanoparticles. Adapted and modified from reference.^[1]^

**Table S1.1:** Relevant process parameters for nanoparticle synthesis via flame spray pyrolysis.

| Metalorganic precursor flow / ml$\cdot$min^-1^ | 2.0 |
| --- | --- |
| Dispersion gas (O_2_) / l$\cdot$min^-1^ | 3.0 |
| Supporting flame (CH_4_) / l$\cdot$min^-1^ | 1.5 |
| Supporting flame (O_2_) / l$\cdot$min^-1^ | 2.0 |
| Sheath gas (O_2_) / l$\cdot$min^-1^ | 5.0 |
| Pressure drop / bar | 2-3 |

### Precursor solutions

**Table S1.2:** Summary of Me-precursors and used organic solvents for the synthesis of MgO-based mixed metal oxide nanoparticles (Me-Mg-O) via FSP. Exemplified on 25 ml batches, the given Me-precursor mass was used to obtain Me-precursor solutions of c(Me) = 0.34 mol∙l^-1^.

| **Mixed metal** | **Me-precursor** | | | **Solvent** |
| --- | --- | --- | --- | --- |
| **oxide system** | **Name** | **Supplier** | **Mass / g** |  |
| Fe-Mg-O | Fe(C5H5)_2_ (98 %) | Sigma-Aldrich/Merck | 1.58 | Xylene |
|  | Bis(cyclopentadienyl)iron(II) |  |  |  |
| In-Mg-O | In(C5H7O2)_2_ (98 %) | Strem Chemicals | 3.5 | Toluene |
|  | Indium(III) acetylacetonate |  |  |  |

## Ceramic manufacturing

**Powder compaction**

A pressing force of 9.81 kN (load of 1 t) was applied (*p* = 74 MPa, using Equation 1 with standard acceleration of gravity *g* = 9.81 m∙s^-^²) and dwelled for 1 min to obtain green compacts in a controlled and reproducible way.

|  | $p\left[ Pa \right]=\frac{F}{A}=\frac{{4\cdot m}_{load}\cdot g}{\pi\cdot r^{2}}$ | Equation S1 |
| --- | --- | --- |

m_load_: applied mass of the press / kg
g: acceleration of gravity / m∙s^-2^r: radius of the compaction tool / m

**Pressureless sintering**

To obtain a smooth ceramic surface the cylindrical green bodies were sandwiched between two alumina plates and placed on top of an alumina crucible in the middle of the furnace. The samples were heated up to the final sintering temperature of 1373 K (ΔT = 5 K∙min^-1^) and dwelled for 2.5 hours (C1373). Afterwards the furnace was switched off and the sample cooled down to room temperature. During the entire sintering protocol, the samples were continuously purged with molecular oxygen (O_2_ 5.0, Q_O2_ = 50 ml∙min^-1^) to remove carbon remnants that arise from the nanoparticle synthesis. An additional sintering step for In^3+^ doped samples with a final sintering temperature of 1673 K (C1673) was conducted (ΔT = 5 K∙min^-1^, t_dwell_ = 2.5 h, Q_O2_ = 50 ml∙min^-1^). **Figure S1.2** shows both sinter programs.


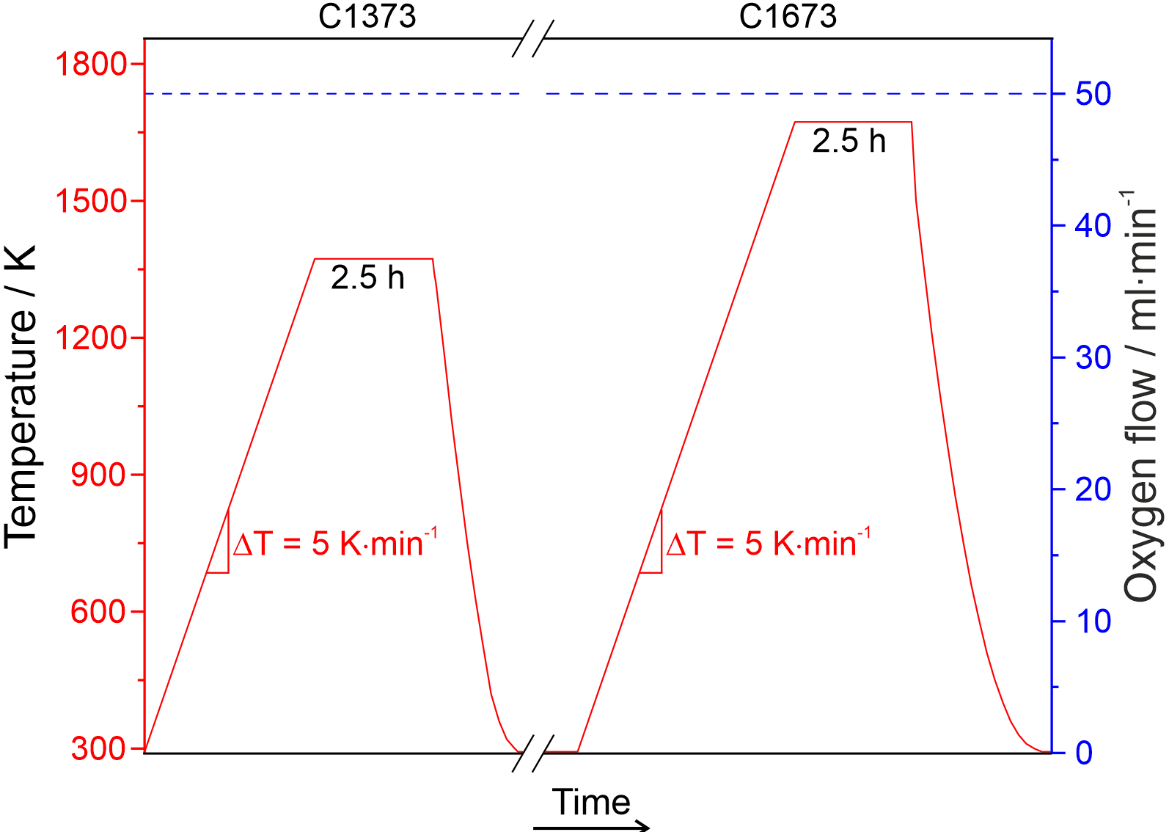


**Figure S1.2:** Schematic illustration of the sinter programs C1373 and C1673.

The residual porosities ϕ of the sintered compacts was calculated geometrically through the weight and volume of the compacts via Equation S2.

|  | $\text{ϕ}\text{ [\%]=}\left( \text{1-}\frac{\text{ρ}_{\text{Specimen}}}{\text{ρ}_{\text{M}\text{e}_{\text{x}}\text{M}\text{g}_{\text{1-x}}\text{O}}} \right)\text{=}\left( \text{1-}\frac{\frac{\text{m}}{\text{π}\text{r}^{\text{2}}\text{h}}}{\text{ρ}_{\text{M}\text{e}_{\text{x}}\text{M}\text{g}_{\text{1-x}}\text{O}}} \right)\text{⋅}\text{100}$ | Equation S2 |
| --- | --- | --- |

To account for the volume fraction of admixed metal ions in the theoretical density values of Me_x_Mg_1-x_O systems we applied the Rule of Mixture, which is described in detail in a recent publication by Schwab et al.^[2]^

## Characterization methods

**TEM specimen preparation**
TEM grids for investigations on particulate samples were prepared by dipping a lacey carbon grid into the powder to investigate structural features and composition of material adhering to the grid.
For microstructural TEM analysis on doped MgO ceramics, the specimens were prepared in a three-step approach. The bulk ceramic sample was cut with a razor blade to a final size of approximately 2 by 2 mm with a thickness of around 700 μm. In the second step, thinning of the specimen down to a thickness of around 20 μm was performed with a precision polishing system (Allied MultiPrepTM). The lubrication free grinding was conducted with diamond coated polymer films with a roughness ranging from 15 μm down to 0.1 μm, rotating at 10-50 rpm. In the last step, the specimen was polished with argon ions inside a precision ion polishing system (PIPS II, Gatan) operating at a background pressure of *p*(Ar) = 4⋅10^-5^ mbar and constant sample cooling down to 273 K. An energy of 6 keV with a glancing angle of ± 5° was applied until the formation of a hole in the specimen’s center with thicknesses below 100 nm around, suitable for high quality TEM investigations. To remove artefacts in the specimen arising from the high-energy argon ions, the damaged regions were removed by a stepwise reduction of the argon beam’s energy (3 keV, 1.5 keV, 0.75 keV, 0.37 keV and 0.1 keV). The last two steps were conducted at a glancing angle of ± 10°.

**Scanning electron microscopy**

Quantification of the chemical composition was conducted with the EDX detector, calibrated with a Cu tape prior to acquisition and at a working distance of 9 mm. Signal integration of counts over the Mg Kα transition line for Mg (integration: 1.12 – 1.37 keV) and respective Kα and Lα transition lines of investigated admixture species (**Table S1.3**) was performed. The elemental composition of 20 different sample spots and/or 3 area scans at the minimum were determined to evaluate the compositional homogeneity and integral dopant concentration levels.

**Table S1.3:** Energy ranges used for the integration over acquired signals during composition analysis with the help of transmission (TEM) and scanning electron microscopy (SEM).

| **Elemental transition**  **line** | **Integration range / keV** | |
| --- | --- | --- |
|  | **TEM** | **SEM** |
| O K$\alpha$ | 0.46-0.59 | 0.37-0.66 |
| Mg K$\alpha$ | 1.16-1.34 | 1.12-1.37 |
| In L$\alpha$ | 3.15-3.41 | 2.78-4.28 |
| Fe K$\alpha$ | 6.24-6.56 | 6.20-7.22 |

To limit charging SEM samples were coated with a few nm thick layer of carbon with a Carsington Carbon Coater 108 carbon/A (2 x 15 s, 4.0 V, background pressure 0.06 mbar) and additionally contacted to the sample holder with copper tape. Image as well as grain size analysis was performed with ImageJ (V1.52a) and the SmartTiff (V3.0) software package from Zeiss.

# Supporting Information – Results

## Me-Mg-O nanoparticle powder characterization

### X-ray diffraction (XRD)


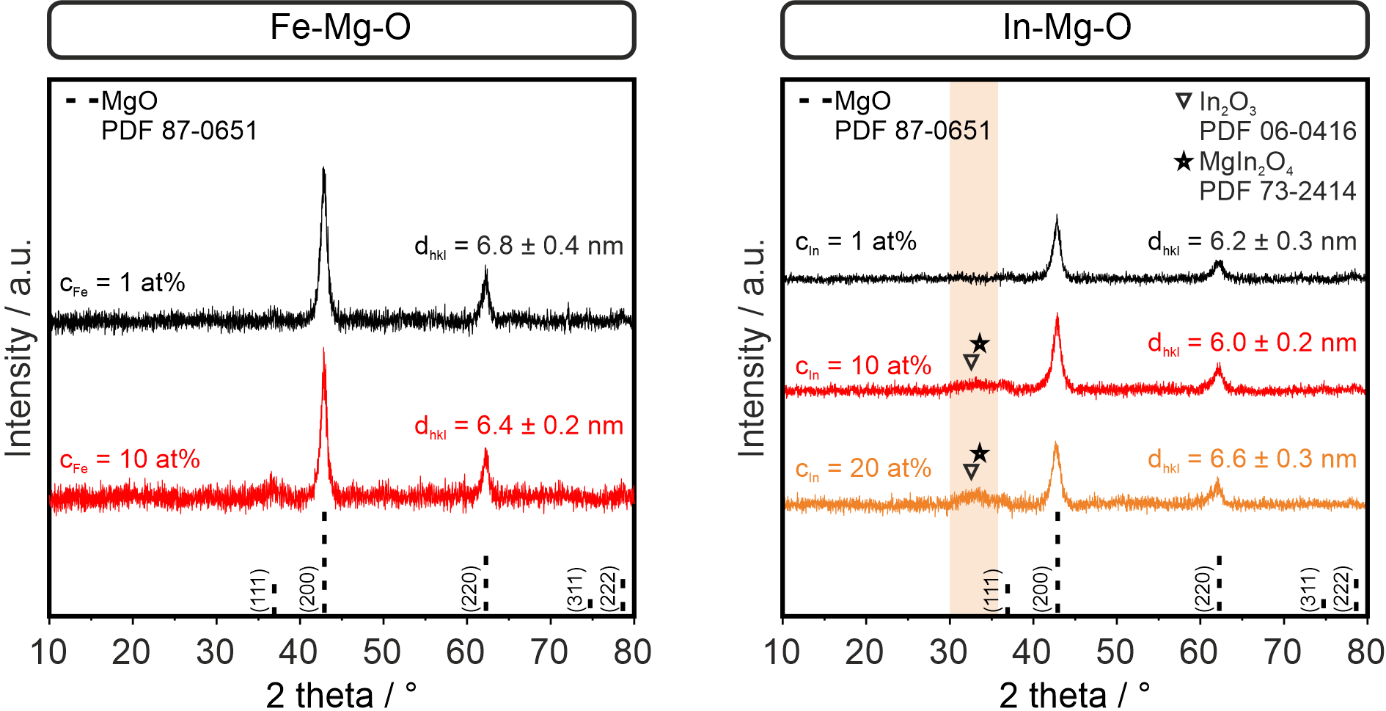


**Figure S2.1:** X-ray diffraction patterns of as synthesized Fe-Mg-O (left) and In-Mg-O (right) nanoparticle powders with admixture concentrations of 1 at%, 10 at% and 20 at%.

**Table S2.1:** Crystallite domain sizes of as synthesized Me-Mg-O nanoparticle powders (c_Me_ = 1 at%, 10 at% and 20 at%) as determined by applying the Scherrer equation to the main reflection of MgO at 2θ = 42.9°.

| **System** | **Crystallite domain size / nm** | | |
| --- | --- | --- | --- |
|  | **1 at%** | **10 at%** | **20 at%** |
| Fe-Mg-O | 6.8 ± 0.4 | 6.4 ± 0.2 | - |
| In-Mg-O | 6.2 ± 0.3 | 6.0 ± 0.2 | 6.9 ± 0.1 |

### Transmission electron microscopy (TEM)


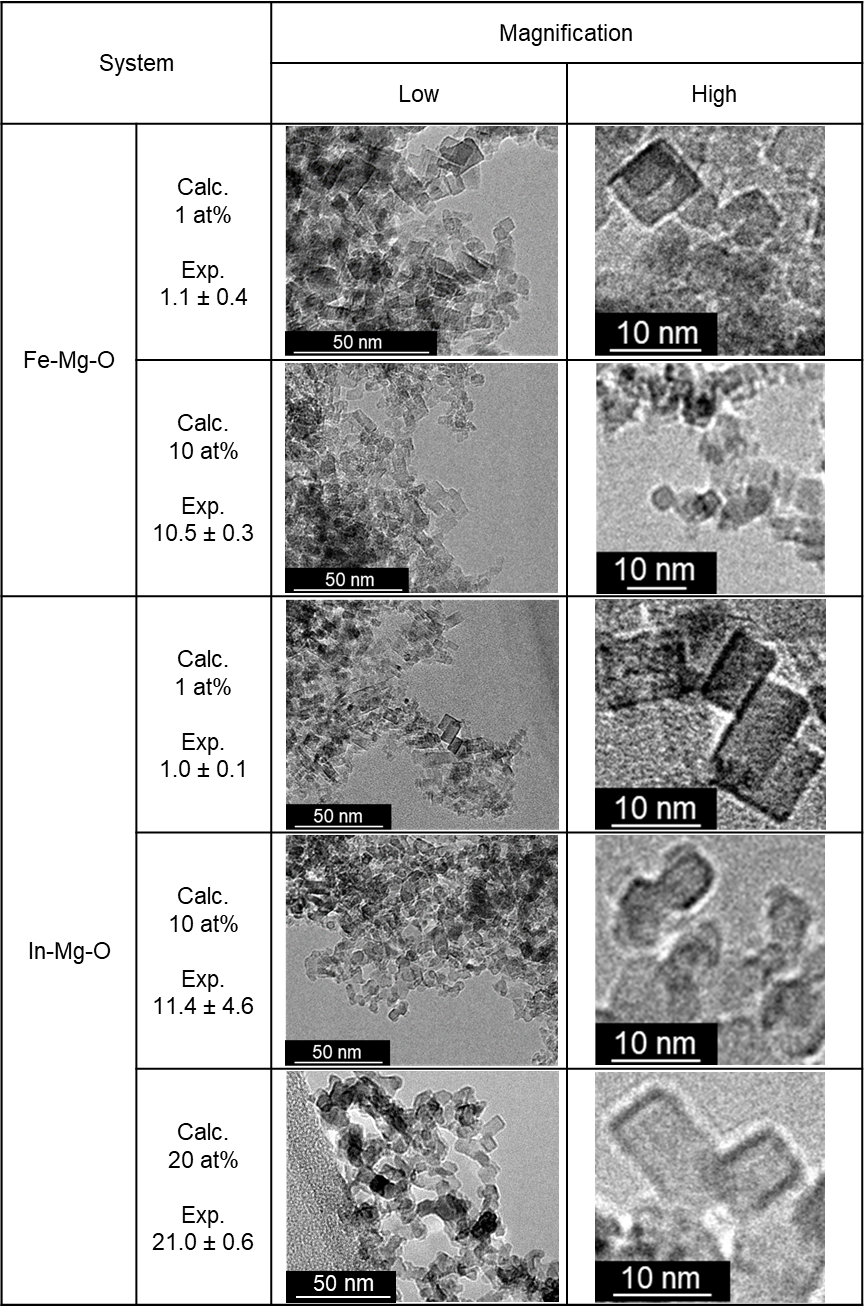


**Figure S2.2:** Low and high magnification TEM images of as synthesized Fe-Mg-O and In-Mg-O nanoparticle powders with c_Me_ = 1 at%, 10 at% and 20 at%.

**
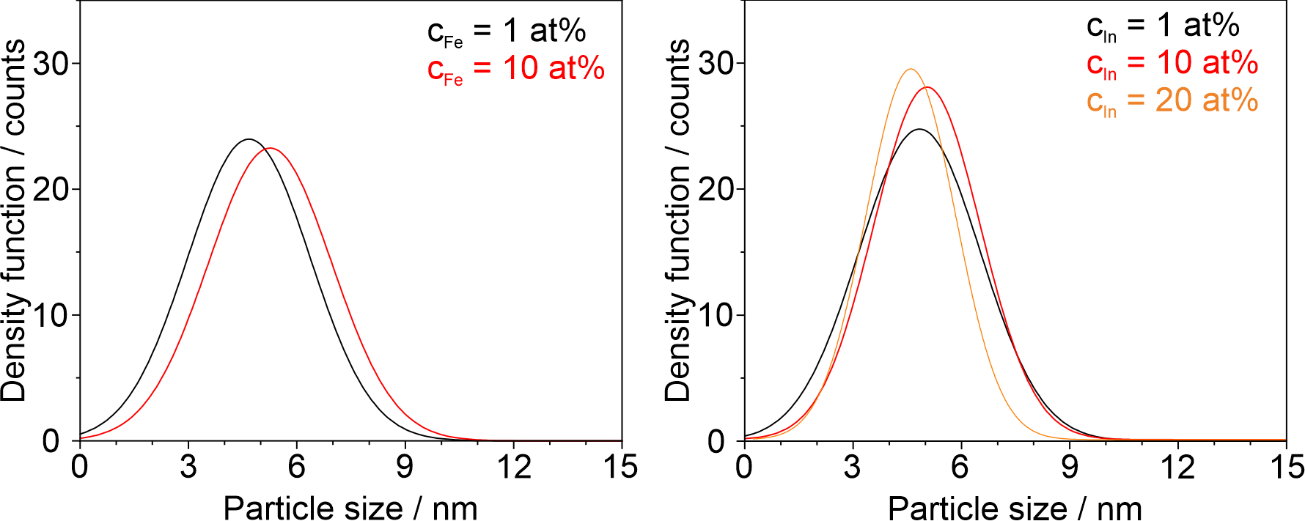
**

**Figure S2.3:** Particle size distributions obtained by particle counting from TEM micrographs, of as synthesized Me-Mg-O nanoparticle powders (Me = Fe, In; c_Me_ = 1 at%, 10 at% and 20 at%)

**Table S2.2:** Median particle sizes of as synthesized Me-Mg-O nanoparticle powders (Me = Fe, In; c_Me_ = 1 at%, 10 at% and 20 at%) obtained by particle counting from TEM micrographs.

| **System** | **Median particle size / nm** | | |
| --- | --- | --- | --- |
|  | **1 at%** | **10 at%** | **20 at%** |
| Fe-Mg-O | 4.6 ± 0.5 | 5.1 ± 0.5 | - |
| In-Mg-O | 4.8 ± 0.5 | 4.9 ± 0.5 | 4.5 ± 0.5 |

## Sintered ceramic characterization


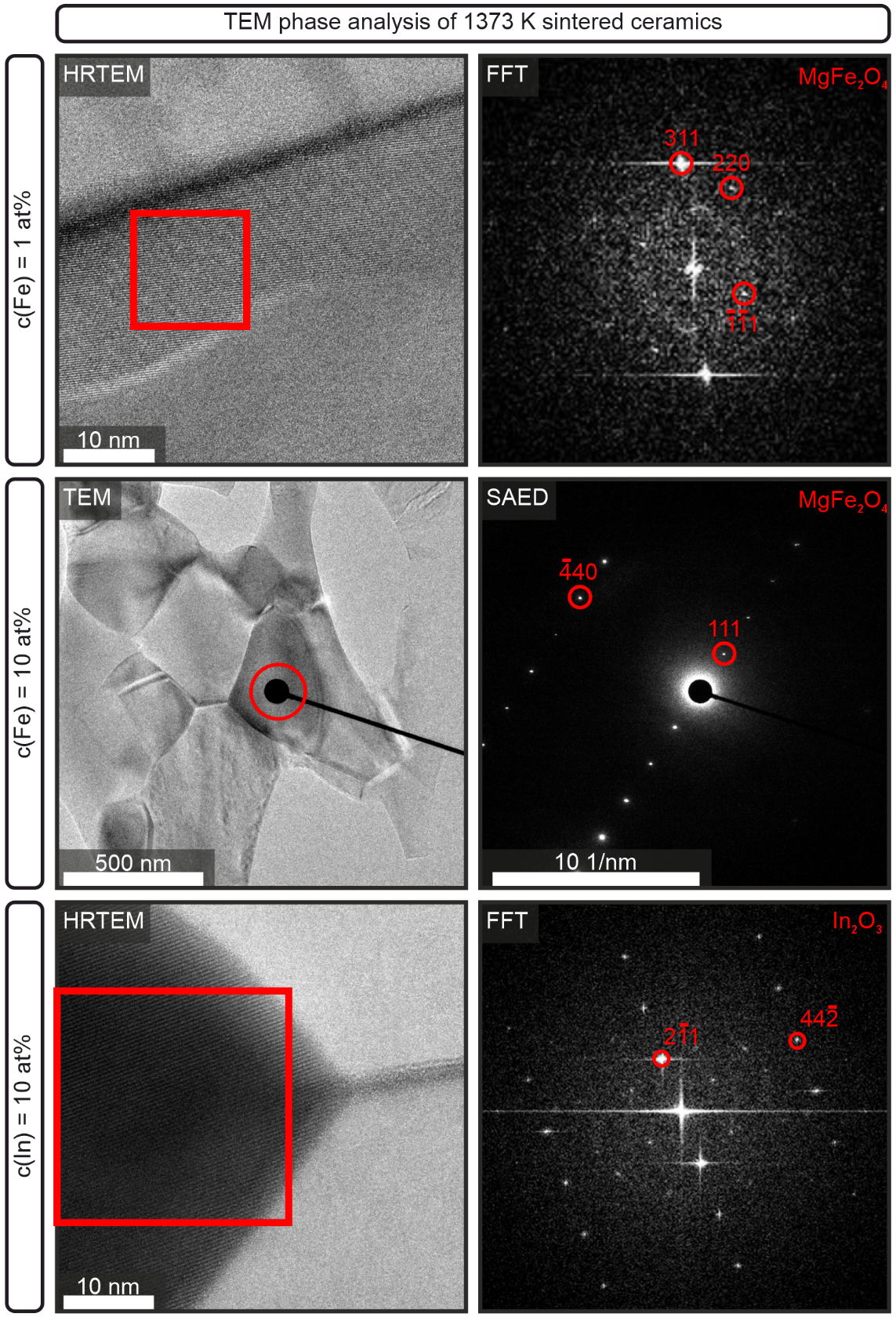


**Figure S2.4:** TEM and HRTEM images with corresponding FFT and SAED images for phase analysis with the transmission electron microscope on Fe-Mg-O (first and second row) and In-Mg-O (third row) ceramics after sintering at 1373 K. Squares and circles within the HRTEM and TEM images indicate characteristic regions that were used for phase identification of MgFe_2_O_4_ and In_2_O_3_.


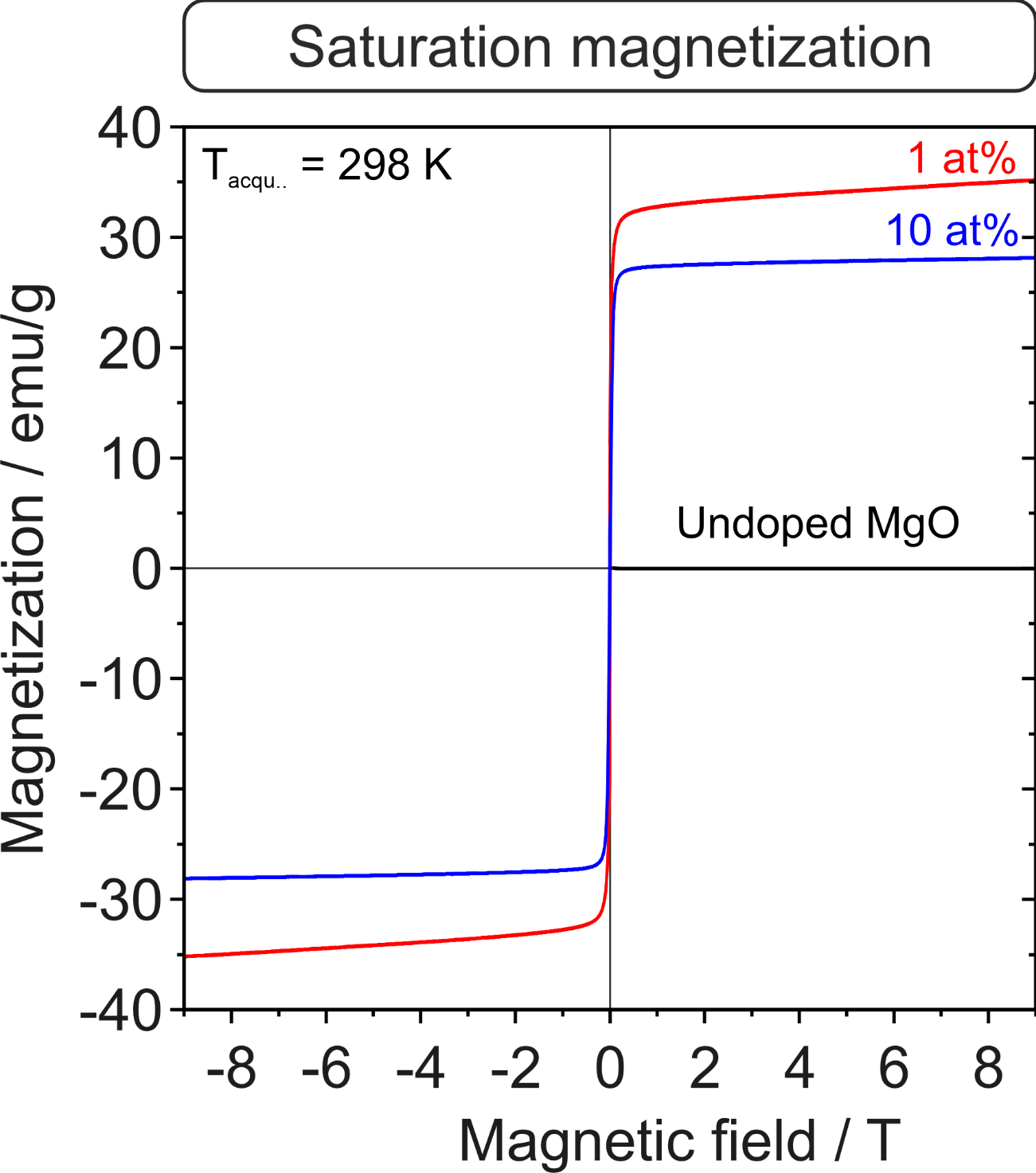


**Figure S2.5:** Saturation magnetization measurements of an undoped MgO ceramic compared to Fe-Mg-O ceramics with nominal Fe-concentrations of 1at% and 10 at%, sintered at 1373 K.

**Table S2.3:** Literature values of the saturation magnetization (M_S_) for MgFe_2_O_4_ structures obtained via different synthesis techniques together with reported crystallite (d_XRD_) and or particle sizes (d_SEM_, d_TEM_).

| Synthesis  method | Crystallite (*d*_XRD_)  and/or particle size (*d*_SEM_, *d*_TEM_)  / nm | *T*_acqu._  / K | Saturation  Magnetization *M*_s_  / emu$\cdot$g^-1^ | Ref. |
| --- | --- | --- | --- | --- |
| **MgFe_2_O_4_ bulk structures** | | | | |
| n.a. | n.a. | RT | 27 | ^[3]^ |
| n.a. | n.a. | 293 | 26.9 | ^[4]^ |
| n.a | n.a. | 3 | 33.4 | ^[5–7]^ |
| **MgFe_2_O_4_ ceramics** | | | | |
| Ball milling and  sintering | *d*_XRD_ = 60  *d*_SEM_ = 8000 | RT | 20.5 | ^[8]^ |
| Self-ignited sol-gel  method and SPS | *d*_TEM_ = 64-170 | 300 | 31-64 | ^[9]^ |
| **MgFe_2_O_4_ nanoparticles** | | | | |
| Combustion | *d*_TEM_ = 10-40 | 300 | 33.8 | ^[10]^ |
| Combustion  (Mg_x_Fe_3-x_O_4_  0.5≤x≤1.5) | *d*_XRD_ = 41-51  *d*_TEM_ = 42-49 | 340 | 25-50 | ^[11]^ |
| Solution  combustion | *d*_XRD_ = 9, d_TEM_ = 11  *d*_XRD_ = 59, d_TEM_ = 58 | RT | 22  31 | ^[12]^ |
| Sol-Gel  (autocombustion) | *d*_XRD_ = 35, d_TEM_ < 100 | RT | 22 | ^[4]^ |
| Microwave-assisted  hydrothermal method | *d*_XRD_ = 3  *d*_TEM_ = 2 | 50  12 | < 20  20 | ^[13]^ |
| Mechanochemical | *d*_XRD_ = 8.5, 20.4, 42, 93 | 3 | 50, 42.2, 37.1, 33.8 | ^[5]^ |
| High energy  ball milling | *d*_XRD_ = 10 | 293  4.5 | 27  36.7 | ^[7]^ |
| Hydrothermal  synthesis | *d*_XRD_ = 11.2, 13.5, 14,  14,14.2 | 300 | 53.9, 65.4, 59.8  64.7, 57 | ^[14]^ |
| Thermal  decomposition | *d*_XRD_ = 6-17 | 290 | 8.1-16.9 | ^[15]^ |
| Co-precipitation | *d*_XRD_ = 6-18 | 300 | <10 | ^[16]^ |
| Electrospun PVP/  MgFe_2_O_4_ | *d*_XRD_ = 15-24 | 293 | 17-31.1 | ^[17]^ |


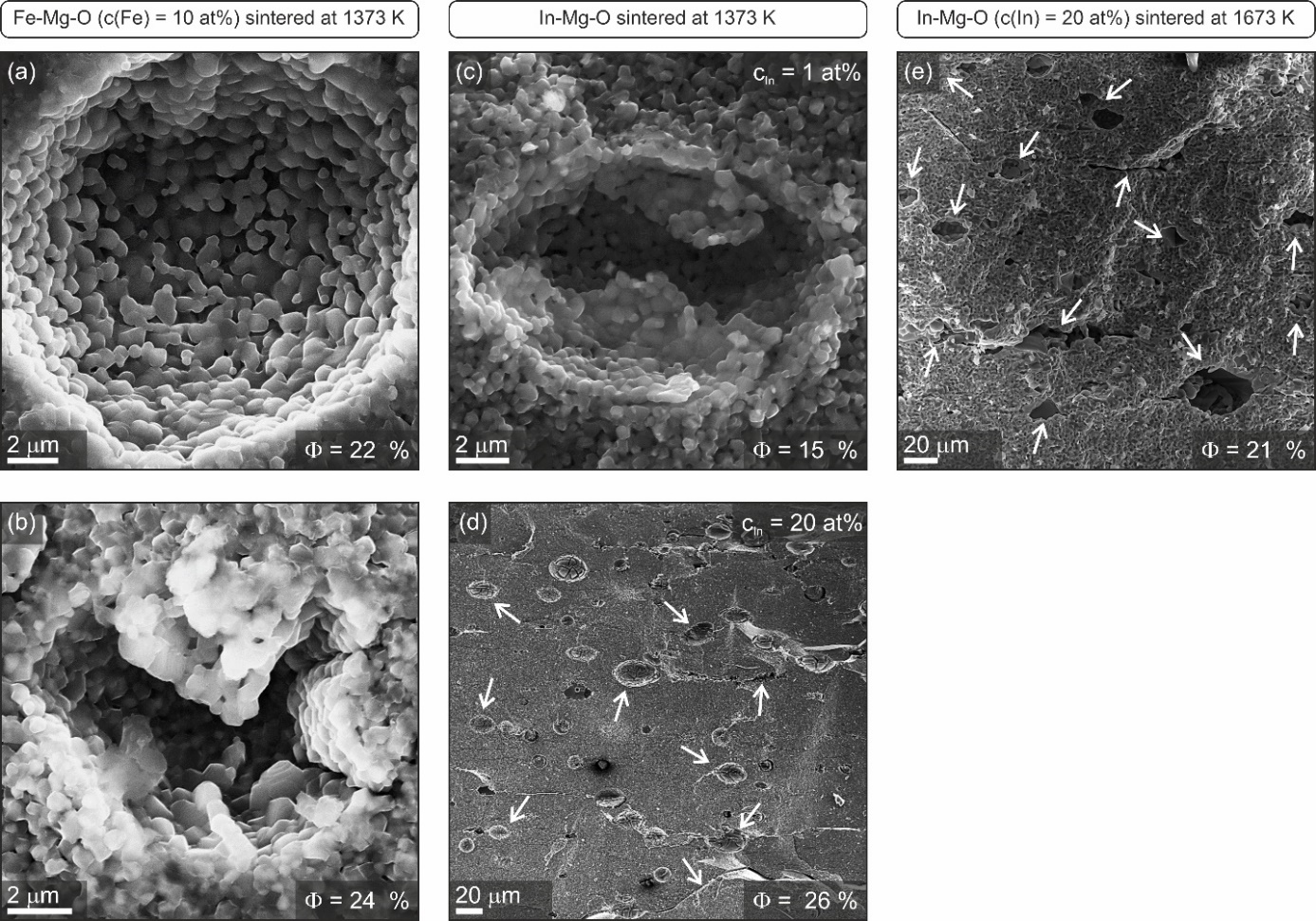


**Figure S2.6:** Secondary electron SEM micrographs obtained from ceramic fracture surface topology (IL-detector) analysis of surface inhomogeneities (pores, voids and cracks). Values in the bottom right corner of the IL-images represent obtained porosity values. *First column (a, b):* Pores in Fe-Mg-O ceramics with a nominal Fe-concentration of 10 at% sintered at 1373 K. *Second column:* 1373 K sintered In-Mg-O ceramics with a nominal In-concentration of 1 at% (c, top) and 20 % (d, bottom) showing pores at high magnification and pores, voids and cracks at low magnification indicated by white arrows. *Third column (e):* Low magnification image of an In-Mg-O (c(In) = 20 at%) ceramic sintered at 1673 K showing pores, voids and cracks indicated by white arrows.


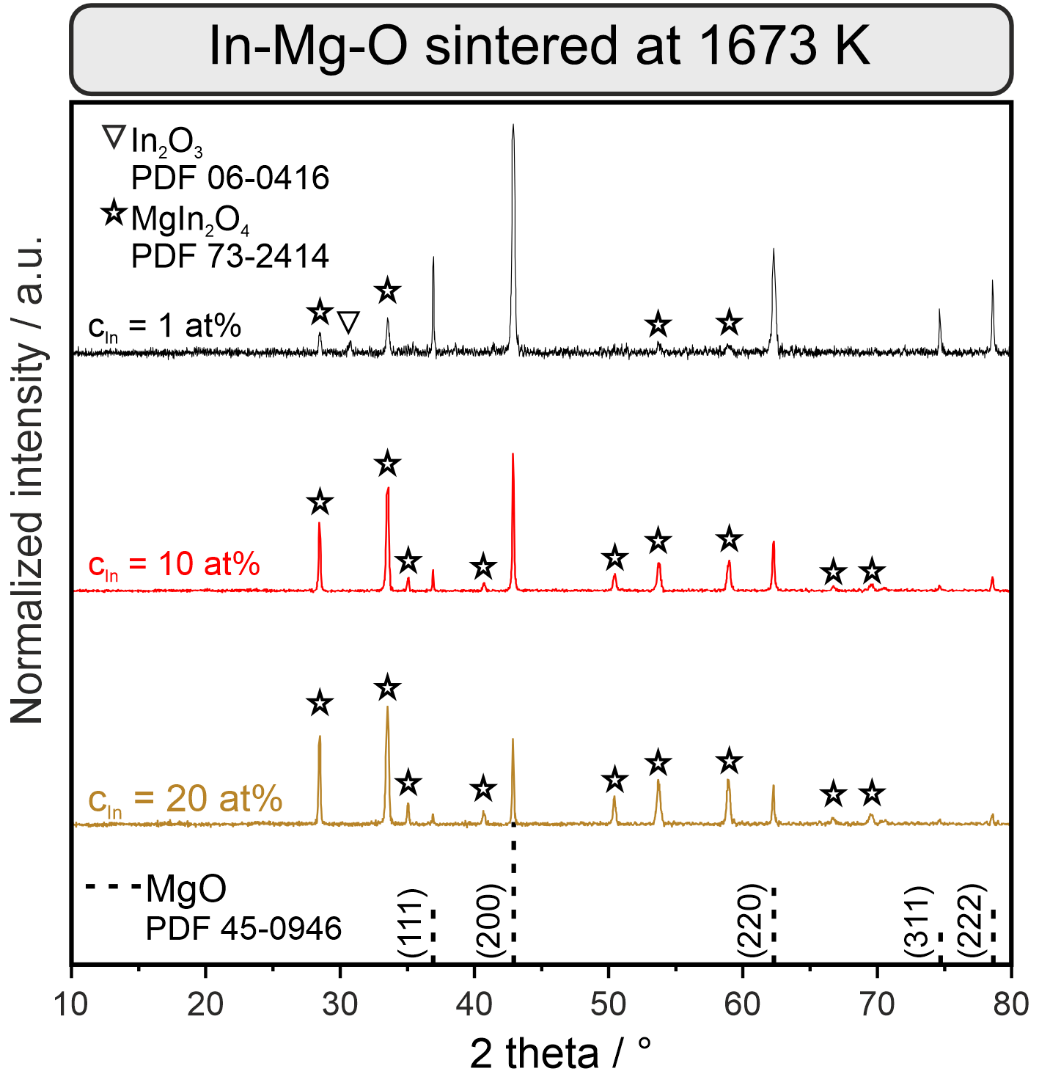


**Figure S2.7:** X-ray diffraction patterns of In-Mg-O ceramics sintered at 1673K with admixture concentrations of 1 at%, 10 at% and 20 at%.


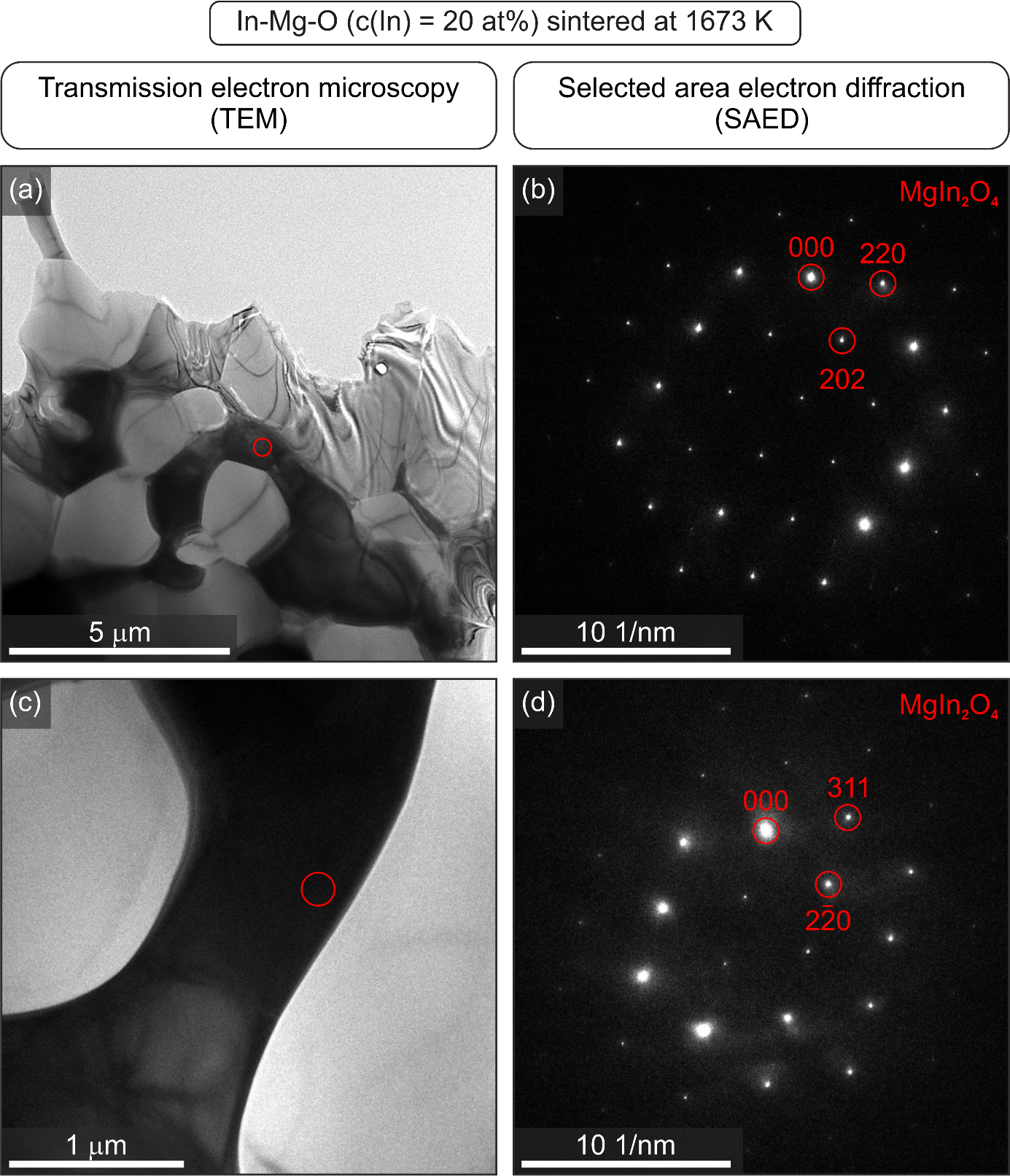


**Figure S2.8:** TEM images (left column a, c) and corresponding SAED images (right column b, d) proving the presence of the MgIn_2_O_4_ spinel phase within the 1673 K sintered In-Mg-O ceramic with an In-concentration of c(In) = 20 at%.

# References

[1] L. Mädler, H. K. Kammler, R. Mueller, S. E. Pratsinis, *J. of Aerosol Sci.* **2002**, *33*, 369.

[2] T. Schwab, K. Aicher, H. Razouq, G. A. Zickler, O. Diwald, *ACS Appl. Mater. Interfaces* **2021**, *13*, 25493.

[3] R. G. Kulkarni, H. H. Joshi, *J. Solid State Chem.* **1986**, *64*, 141.

[4] A. Pradeep, P. Priyadharsini, G. Chandrasekaran, *J. Magn. Magn. Mater.* **2008**, *320*, 2774.

[5] V. Šepelák, I. Bergmann, D. Menzel, A. Feldhoff, P. Heitjans, F. J. Litterst, K. D. Becker, *J. Magn. Magn. Mater.* **2007**, *316*, e764-e767.

[6] V. Šepelák, A. Feldhoff, P. Heitjans, F. Krumeich, D. Menzel, F. J. Litterst, I. Bergmann, K. D. Becker, *Chem. Mater.* **2006**, *18*, 3057.

[7] V. Šepelák, D. Baabe, D. Mienert, F. J. Litterst, K. D. Becker, *Scr. Mater.* **2003**, *48*, 961.

[8] M. Manikandan, P. Manimuthu, C. Venkateswaran in *AIP Conference Proceedings*, AIP Publishing LLC, **2014**, pp. 194–196.

[9] M. Penchal Reddy, R. A. Shakoor, A. Mohamed, M. Gupta, Q. Huang, *Ceram. Int.* **2016**, *42*, 4221.

[10] V. M. Khot, A. B. Salunkhe, N. D. Thorat, M. R. Phadatare, S. H. Pawar, *J. Magn. Magn. Mater.* **2013**, *332*, 48.

[11] A. Franco, T. E. Pereira Alves, E. C. de Oliveira Lima, E. Da Silva Nunes, V. Zapf, *Appl. Phys. A* **2009**, *94*, 131.

[12] S. Da Dalt, A. S. Takimi, T. M. Volkmer, V. C. Sousa, C. P. Bergmann, *Powder Technol.* **2011**, *210*, 103.

[13] S. Verma, P. A. Joy, Y. B. Khollam, H. S. Potdar, S. B. Deshpande, *Mater. Lett.* **2004**, *58*, 1092.

[14] J. Nonkumwong, S. Ananta, P. Jantaratana, S. Phumying, S. Maensiri, L. Srisombat, *J. Magn. Magn. Mater.* **2015**, *381*, 226.

[15] B. Aslibeiki, G. Varvaro, D. Peddis, P. Kameli, *J. Magn. Magn. Mater.* **2017**, *422*, 7.

[16] Q. Chen, Z. J. Zhang, *Appl. Phys. Lett.* **1998**, *73*, 3156.

[17] S. Maensiri, M. Sangmanee, A. Wiengmoon, *Nanoscale Res. Lett.* **2008**, *4*, 221.
